# Grape Powder Improves Age-Related Decline in Mitochondrial and Kidney Functions in Fischer 344 Rats

**DOI:** 10.1155/2016/6135319

**Published:** 2016-07-27

**Authors:** Indira Pokkunuri, Quaisar Ali, Mohammad Asghar

**Affiliations:** ^1^Heart and Kidney Institute, Pharmacological and Pharmaceutical Sciences, College of Pharmacy, University of Houston, Houston, TX 77204, USA; ^2^University of Oklahoma Health Sciences Center, Oklahoma City, OK 73104, USA

## Abstract

We examined the effects and mechanism of grape powder- (GP-) mediated improvement, if any, on aging kidney function. Adult (3-month) and aged (21-month) Fischer 344 rats were treated without (controls) and with GP (1.5% in drinking water) and kidney parameters were measured. Control aged rats showed higher levels of proteinuria and urinary kidney injury molecule-1 (KIM-1), which decreased with GP treatment in these rats. Renal protein carbonyls (protein oxidation) and gp^*91phox*^-NADPH oxidase levels were high in control aged rats, suggesting oxidative stress burden in these rats. GP treatment in aged rats restored these parameters to the levels of adult rats. Moreover, glomerular filtration rate and sodium excretion were low in control aged rats suggesting compromised kidney function, which improved with GP treatment in aged rats. Interestingly, low renal mitochondrial respiration and ATP levels in control aged rats were associated with reduced levels of mitochondrial biogenesis marker MtTFA. Also, Nrf2 proteins levels were reduced in control aged rats. GP treatment increased levels of MtTFA and Nrf2 in aged rats. These results suggest that GP by potentially regulating Nrf2 improves aging mitochondrial and kidney functions.

## 1. Introduction

Aging is a progressive degenerative process that adversely affects different organ functions. Among these, age associated changes in the kidney are the most dramatic [1]. Kidney aging, also termed as renal senescence, involves multifold anatomical and functional changes. Age-related anatomical changes in the kidney include presence of sclerotic glomeruli and interstitial fibrosis [2], while functional changes include decreases in creatinine clearance and elevated levels of urinary proteins (proteinuria) [3–5].

Mitochondria, the chief source of cellular energy currency ATP, are reported to decay during aging causing mitochondrial respiratory failure [6, 7] and mitochondrial myopathy [8] and are also involved in the generation of reactive oxygen species (ROS) [9]. ROS-mediated kidney damage and functional kidney impairment are well described during aging [10–13]. Relevant to this, animal studies have suggested a causal role for oxidative stress (ROS) in age-related hypertension and also in kidney damage during aging [14, 15]. Thus, the link between oxidative stress, mitochondrial dysfunction, kidney damage, and functional impairment during aging is strong.

Grape powder [16–20] recently has received much attention with regard to its health benefits. However, whether grape powder has any beneficial effects on aging kidney and its impact on improving mitochondrial functions are not known. Therefore, the protective effects and mechanism of GP-mediated improvement in aging kidney and mitochondrial functions have been examined in this study using a rat aging model, Fischer 344 rats.

## 2. Materials and Methods

### 2.1. Animals

Adult (1-month) and aged (21-month) male Fischer 344 (F344) rats raised by Taconic Farms, Inc. (Hudson, NY), were purchased from the National Institute on Aging. The rats were housed in plastic cages in the University of Houston animal care facility and were used as per the National Institutes of Health guidelines and approved protocols by the University of Houston Animal Care and Use Committee. The rats were acclimatized in the animal facility for 1 week before any treatment was undertaken.

### 2.2. Freeze-Dried Grape Powder

Freeze-dried grape powder (GP) was obtained from California Table Grape Commission (California Table Grape Commission, Fresno, CA). The composition of the grape powder includes fresh red, green, and black California grapes (seeded and seedless varieties) that have been frozen, ground with food quality dry ice, freeze-dried, and reground using Good Manufacturing Practices for food products throughout. The powder was processed and stored to preserve the integrity of the biologically active compounds found in fresh grapes. Detailed composition of the grape powder was included in Table 1(a). The grape powder was hygroscopic and was provided in small, sealed packets. Upon receipt, it was stored at −80°C in moisture proof containers until used. Fresh solution of 1.5% grape powder in tap water was made every day to feed the rats. This grape powder dose was carefully chosen after conducting pilot dose-response studies as the selected dosage showed most pronounced effects on oxidative stress and blood pressure in these rats [18, 20].

### 2.3. Grape Powder Treatment

Adult and aged rats were divided into control and grape powder groups. GP groups of adult and aged rats were provided with 1.5% freeze-dried grape powder in drinking water for 6 weeks as we reported earlier [20]. Control groups of adult and aged rats received only drinking water (without GP) for 6 weeks. Thereafter, rats were anesthetized to determine different parameters as detailed below. Food and water supplemented without and with GP were provided to rats* ad libitum*.

### 2.4. Animal Surgery

Animals were anesthetized with Inactin® (100 mg/kg, i.p.) and tracheotomy was performed to facilitate spontaneous breathing followed by cannulating left carotid artery with P-50 tubing using our previously published procedures [15]. Briefly, the tubing was connected to a pressure transducer, which was connected to a blood pressure recording device (Grass Polygraph, model 7D; Grass Instrument, Quincy, MA). After the stabilization of 15 min, blood pressure was recorded for 30 min. Thereafter, left jugular vein was catheterized with PE-50 tubing and 50 *μ*L of blood collected through carotid artery. Left ureter was catheterized with PE-10 tubing, saline was infused through jugular vein (1% body weight/hr, volume expansion), and two 30 min urine samples were collected through ureter. The parameters obtained for two urine collections were averaged and considered for data interpretations. After collecting second urine, 50 *μ*L of blood from carotid artery was collected, and plasma was separated by centrifugation and stored at −80°C for further analyses.

Second set of control and GP treated adult and aged rats were anesthetized as above, midline abdominal incision made with a scalpel blade and bladder urine obtained with syringe for biochemical studies. Kidneys were isolated and kept in cold saline. Superficial kidney cortex (rich in proximal tubules) was isolated and nuclear and cytosolic fractions were made on the same day (described below). All other samples were aliquoted and frozen at −80°C until further use.

### 2.5. Sodium and Creatinine Measurements

Ureter urine and plasma samples were used to measure sodium and creatinine. Sodium concentrations were measured using atomic absorption spectrometer (AAnalyst 400, Perkin Elmer, Waltham, MA) as described before [15]. Creatinine levels were determined using a commercially available assay kit (catalog number K625-100; BioVision, Mountain View, CA) as published [25]. GFR, as a function of creatinine clearance, was determined using the following equation: urinary volume × urinary creatinine ÷ plasma creatinine.

### 2.6. Bladder Urine Analyses

Commercially available kits were used to measure kidney injury molecule (KIM-1) (R & D Systems Inc. Minneapolis, MN) and protein (Thermo Scientific, Rockford, IL).

### 2.7. Nuclear and Cytosolic Fraction Isolation

A commercially available kit (Thermo Scientific, Rockford, IL) was employed to purify nuclear and cytosolic fractions from superficial cortex as described [26].

### 2.8. Real-Time PCR

Real-time PCR was performed as described [25]. Briefly, total RNA from superficial cortical tissues was purified using a kit based method (Qiagen Inc., Valencia, CA) and used to synthesize cDNA using Advantage RT for PCR kit (Clontech Inc., Mountain View, CA). The cDNA obtained was further diluted 5 times and used (10 *μ*L) in the real-time PCR reaction with TaqMan rat specific primers for MtTFA (assay ID Rn00580051_m1) from Applied Biosystems (Life Technologies, Grand Island, NY). 18S rRNA was run in parallel as internal control and used to normalize the data. Data were compared using Delta-Delta Ct method.

### 2.9. Mitochondrial Respiration

Respiration was measured using Oxygraph-2K (Oroboros Instruments Corp. Austria). Briefly, zero and air calibrations were performed with water followed by calibration with 2 mL MiR05 medium (mM: 0.5 EGTA, 3 MgCl_2_, 60 potassium lactobionate, 20 taurine, 10 KH_2_PO_4_, 20 HEPES, 110 sucrose, and 1 g/L fatty acid free BSA). The chamber was allowed to equilibrate with an ambient gas phase (air) at 37°C with a stirrer speed of 750 rpm for >30 minutes to allow air saturation of the respiration medium. Superficial cortical tissue homogenates were made in MiR05 using SG3 shredder (Pressure Biosciences Inc., South Easton, MA). Mitochondrial respiration was measured using 100 *μ*g homogenate proteins [27, 28].

### 2.10. Oxidative Stress Markers

Cortical tissue homogenates were used to determine protein oxidation using Oxiblot protein oxidation Kit (EMD Millipore, Billerica, MA) and gp^*91phox*^-NADPH oxidase by Western blotting.

### 2.11. Western Immunoblotting

Western immunoblotting in cytosolic and nuclear fractions and tissue homogenates were performed using standard methods as described previously [29]. Tissue homogenates were made in lysis buffer (mM: 20 Tris-HCl (pH 7.5), 150 NaCl, 1 Na_2_EDTA, 1 EGTA, 1% Triton, 2.5 sodium pyrophosphate, 1 beta-glycerophosphate, 1 Na_3_VO_4_, 1 *μ*g/mL leupeptin, and protease inhibitor cocktail) as described [29]. Protein concentrations in the cytosolic and nuclear fractions and in homogenates were determined by BCA protein assay kit (Thermo Scientific, Rockford, IL). Equal amounts of proteins (20 *μ*g) from cytosolic and nuclear fractions and from tissue homogenates were used for Western immunoblotting using specific antibodies for Nrf2 (Abcam, Cambridge, MA), gp^*91phox*^-NADPH oxidase (Cell Signaling Tech., Beverly, MA), *β*-actin (EMD Millipore, Billerica, MA), and lamin B (Cell Signaling Technology, Inc., Beverly, MA). All the respective HRP conjugated secondary antibodies were from Santa Cruz Biotechnology, Inc. Imaging and quantification of protein bands were performed using G:BOX software (Syngene, Frederick, MD). The protein band density for homogenate and cytosolic samples was normalized with protein loading control, *β*-actin, and that of nuclear fractions with lamin B.

### 2.12. ATP Determination Assay

Quantitative determination of ATP was carried out using a commercially available bioluminescence assay kit (Life Technologies, Grand Island, NY) following manufacturer's instructions. The assay relies on luciferase's requirement for ATP to produce bioluminescent light. Tissue homogenates (10 *μ*g) prepared in MiR05 buffer (mentioned under mitochondrial respiration above) were used to measure ATP concentration. A standard curve was run in parallel using 1–1000 nM ATP in a reaction mixture that contains 0.5 mM D-luciferin and 15 *μ*g of recombinant luciferase. The reaction was followed for 10 min and the light produced read at 560 nm. ATP levels in tissue samples were determined using the generated ATP standard curve.

### 2.13. Data Analysis

Data are presented as mean ± SEM. One-way ANOVA followed by Newman-Keuls* post hoc* test was applied to achieve significance using statistical analysis software (Graphpad Prism, San Diego, CA). The minimum level of significance was considered at *P* < 0.05.

## 3. Results

Detailed chemical composition of freeze-dried grape powder is shown in Table 1(a) that includes flavonoids, phenolic compounds, anthocyanins, and resveratrol. Table 1(b) shows nontoxicity of the grape powder constituents except for resveratrol which resulted in renal toxicity at high concentrations when used alone.

**Figure 1:**
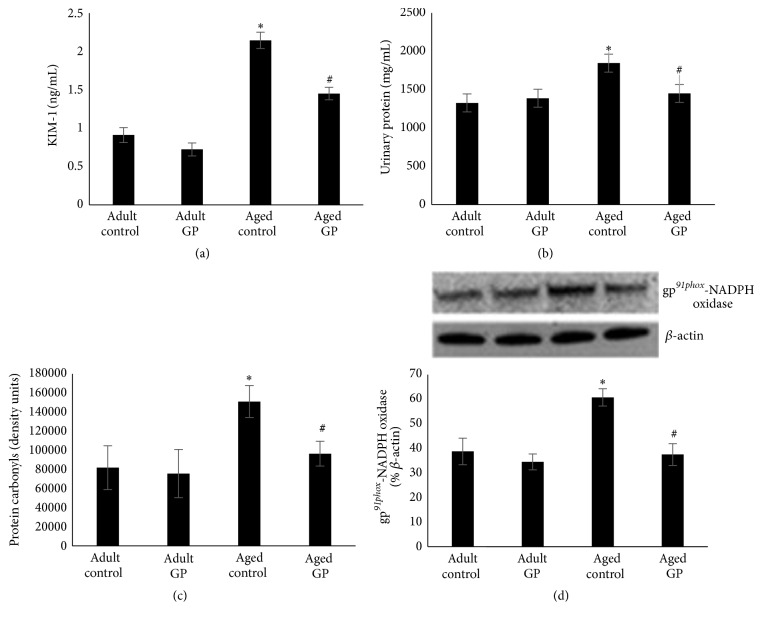
Kidney injury (urinary KIM-1 and protein) and oxidative stress (protein carbonyls and NADPH oxidase) markers in control and grape power (GP) treated adult and aged rats. KIM-1 and protein levels were measured in the urine (a, b). Protein carbonyls and gp^*91phox*^-NADPH oxidase were determined in renal tissues (c, d). KIM-1 (a), protein (b), and protein carbonyls (c) were determined using kit based assay system. gp^*91phox*^-NADPH oxidase (d) was determined by Western blotting. (d) Upper panel: representative blots for NADPH oxidase and *β*-actin, protein loading control. Lower panel: quantification of protein bands. Results are mean ± SEM. *n* = 7-8 rats. *P* < 0.05 from adult (*∗*) and aged control (#) rats.

As shown in Figure 1, kidney injury markers, namely, proteinuria (Figure 1(a)) and urinary KIM-1 (Figure 1(b)) as well as oxidative stress markers such as protein oxidation (Figure 1(c)) and gp^*91phox*^-NADPH oxidase (Figure 1(d)), were elevated in control aged compared to adult rats. Proteinuria, KIM-1, protein oxidation, and gp^*91phox*^-NADPH oxidase levels decreased with GP treatment in aged rats (Figures 1(a)–1(d)). Mean blood pressure was similar in adult (control versus GP: 100 ± 2 versus 97 ± 2 mmHg) and aged (control versus GP: 92 ± 3 versus 99 ± 8 mmHg) rats irrespective of GP treatment.

**Figure 2:**
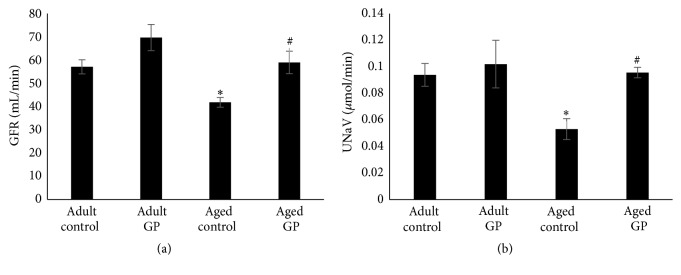
Kidney function in control and grape power (GP) treated adult and aged rats. Kidney function was measured as creatinine clearance, an index of glomerular filtration rate (GFR) (a) and volume expansion-induced natriuresis, UNaV (b) as detailed in the Methods. Results are mean ± SEM. *n* = 7-8 rats. *P* < 0.009 from adult (*∗*) and aged control (#) rats.

**Figure 3:**
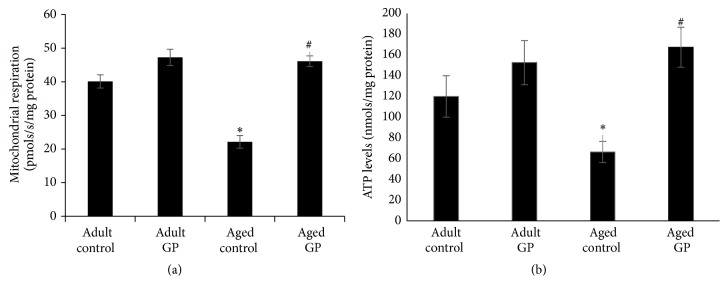
Renal mitochondrial function in control and grape power (GP) treated adult and aged rats. Mitochondrial function was determined by measuring oxygen consumption (respiration) (a) and ATP levels (b) in renal tissues. Oxygen consumption (a) and ATP levels (b) were measured using Oroboros instrument and kit based assay system, respectively (details in the Methods). Results are mean ± SEM. *n* = 7-8 rats. *P* < 0.05 from adult (*∗*) and aged control (#) rats.

Kidney function, as determined by GFR and sodium excretion, was reduced in control aged compared to adult rats (Figures 2(a) and 2(b)). GP treatment increased GFR and sodium excretion in aged rats (Figures 2(a) and 2(b)). Mitochondrial function, as determined by oxygen consumption and ATP levels, was also reduced in aged compared to adult control rats (Figures 3(a) and 3(b)), which increased with GP treatment in aged rats (Figures 3(a) and 3(b)).

**Figure 4:**
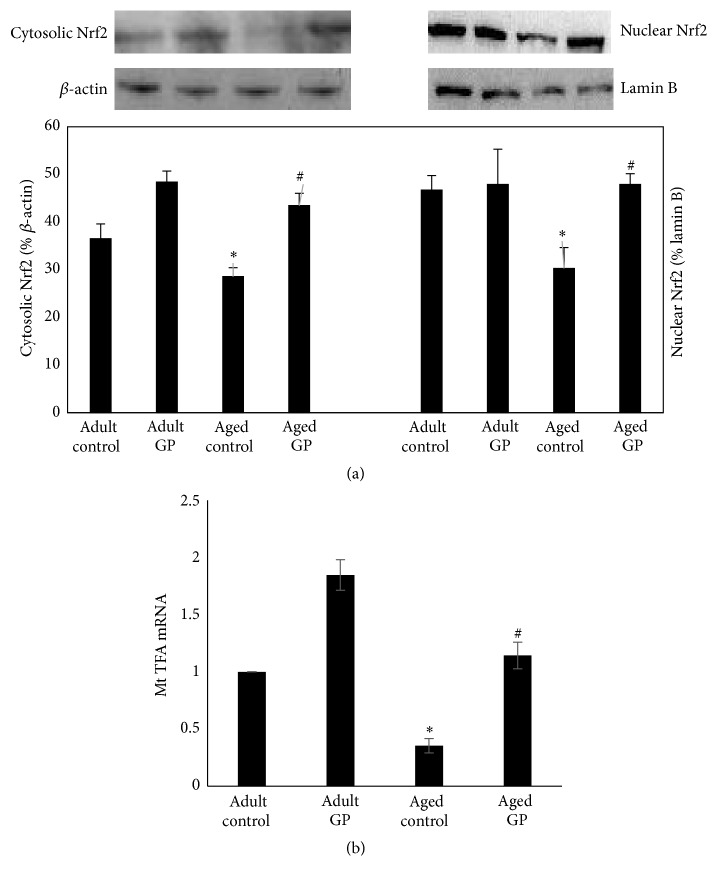
Nrf2 (a) and mitochondrial biogenesis marker MtTFA (b) in the kidney of control and grape power (GP) treated adult and aged rats. (a) Nrf2 in cytosolic and nuclear fractions was determined by Western blotting. Upper panel: representative blots. *β*-actin and lamin B were used as protein loading controls for cytosolic and nuclear fractions, respectively. Lower panel: quantification of protein bands. (b) MtTFA mRNA levels were determined by qPCR. Details for (a) and (b) are in the Methods. Results are mean ± SEM. *n* = 7-8 rats. *P* < 0.0001 from adult (*∗*) and aged control (#) rats.

Further, cytosolic and nuclear levels of Nrf2 were decreased in control aged compared to adult rats, which increased with GP treatment in aged rats (Figure 4(a)). The mRNA levels of mitochondrial transcription factor A (MtTFA) were reduced in aged compared to adult rats (Figure 4(b)). GP treatment increased MtTFA mRNA levels in aged rats (Figure 4(b)).

## 4. Discussion

The current study demonstrates that GP treatment improved kidney function as determined by GFR and natriuresis (sodium excretion) in aged rats. This could be attributed to the antioxidant property of GP [18, 30, 31]. GP while improving kidney function also decreased age-related increase in oxidative stress in these rats. Furthermore, GP reduced age-related kidney injury as kidney injury markers, namely, proteinuria and KIM-1 levels, decreased with GP treatment in aged rats.

In order to investigate the mechanism(s) potentially responsible for GP-mediated improvement in aging kidney function, we focused on mitochondrial mechanisms. Mitochondria are critical for the maintenance of normal cellular functions and are reported to decay during aging process and become a major source of cellular oxidative stress burden [32]. We observed that aging kidneys exhibited reduced mitochondrial function which was associated with increased oxidative stress. Furthermore, increased levels of gp91-phox subunit of NADPH oxidase were noted in the aging kidneys. NADPH oxidase is known to generate superoxide and potentially contribute to age-related increase in oxidative stress. It is likely that aging mitochondria (reduced function)-mediated ROS generation contributes to gp91-phox overexpression in the aging kidneys. This postulation is based on our observation that GP, while improving mitochondrial function and reducing oxidative stress burden, also decreased gp91-phox levels in the aging kidneys.

Mitogenesis (mitochondrial biogenesis) is known to regulate mitochondrial function [33, 34]. It is likely that mitogenesis is altered and affects mitochondrial function during aging. This is an attractive postulation considering the following observations. Mitogenesis marker MtTFA levels were decreased in the aging kidneys, which was associated with reduced mitochondrial respiration and ATP levels in the kidneys of aged rats. Interestingly, GP treatment, while increasing MtTFA levels, also improved renal mitochondrial respiration as well as ATP levels in aged rats. These are important observations indicating that reduced mitogenesis contributes to age-related decline in mitochondrial and kidney functions. And GP via mitogenesis potentially improves mitochondrial and kidney functions in aging rats.

Although PGC-1*α* (peroxisome proliferator-activated receptor-gamma coactivator-1*α*) is considered to be the master regulator of mitochondrial biogenesis [35–37], we did not find any change in the levels of PGC-1*α* with GP in adult and aged rats (data not shown). This negates role of PGC-1*α* in GP-mediated improvement in mitogenesis in the aging kidneys.

Contrary to this, Nrf2 (nuclear factor erythroid 2 related factor) seems to be involved as we found increased levels of Nrf2 in GP treated aged rats. Traditionally Nrf2 is viewed as a regulator of antioxidant defense [38]. New role of Nrf2 as a regulator of mitochondrial function is emerging [39]. For example, loss of Nrf2 is linked to depolarization of mitochondria, decreased levels of cellular ATP, and impaired mitochondrial respiration in murine neurons and embryonic fibroblast [40]. Hence, it is likely that the beneficial effects of grape powder are mediated via inducing Nrf2 activity which in turn increases mitogenesis and keeps mitochondrial function intact thus contributing to improvement in aging mitochondrial and kidney functions.

## 5. Conclusions

Grape powder, at 1.5% dose used in the current studies, was used in our earlier published work and no toxicity was observed throughout the treatment period [18]. Grape powder included in this study was prepared from whole grapes and contains polyphenols including resveratrol, flavans, flavanols, anthocyanins, and simple phenols. From this complex mixture, it remains to be determined if one particular component or a combination of components is indispensable in ameliorating aging mitochondrial and kidney functions. But, toxicity was not reported for any of the individual components of grape powder except for resveratrol which caused renal toxicity when used alone [24]. And, grape skin alone failed to reduce oxidative stress burden in obese mice [41]. These reports suggest that perhaps one constituent is not sufficient but whole grapes with all their constituents, as used in this study, are needed to exert beneficial effects. Therefore, it can be suggested that grape powder renders its protection to aging mitochondrial and kidney functions by activating antioxidant defense systems such as Nrf2 and that grape powder intervention might prove beneficial in restoring age-related mitochondrial and kidney dysfunctions. Also, it is proposed that it may be undertaken as combination therapy to treat mitochondrial diseases such as Leigh's syndrome [42, 43] and Kearns-Sayre syndrome [44].

## Figures and Tables

**Table 1a:** (a) Chemical composition of freeze-dried grape powder (California table grape commission)

Compounds	Total	Individual
*Catechins*	22.06 mg/kg	
Catechin		13.7 mg/kg
Epicatechin		8.36 mg/kg
*Anthocyanins*	709.8 mg/kg	
Peonidin		232.2 mg/kg
Cyanidin		156.2 mg/kg
Malvidin		321.4 mg/kg
*Flavonols*		
Quercetin		148.7 mg/kg
Kaempferol		11.61 mg/kg
Isorhamnetin		23.9 mg/kg
*Stilbenes*		
Resveratrol		1.51 mg/kg
*Total polyphenols*		383 mg/100 g

**Table 1b:** (b) Toxicology of grape powder constituents

Compound	Dose (per kg body wt/day)	Duration	Toxicity
Direct catechins	≤2500 mg	Chronic (90 days)	Nontoxic [21]
Anthocyanins	≤2000 mg	Chronic (90 days)	Nontoxic [22]
Flavonoids	≤5000 mg	Acute (24 hrs)	Nontoxic [23]
Resveratrol	≤3000 mg	4 weeks	Toxic [24]
